# Clinical and epidemiological profiles of confirmed malaria cases in the state of Pernambuco, Brazil, from 2001 to 2022

**DOI:** 10.1590/0037-8682-0038-2025

**Published:** 2025-12-15

**Authors:** Diego Lins Guedes, Helena Maria Ramos Guimarães, Maria Clara Galindo Padilha, Maria Cecília Pereira Pinto, Marina Rafaelly Nascimento da Silva, Eduardo Vinicius de Oliveira Andrade

**Affiliations:** 1 Universidade de Pernambuco, Faculdade de Ciências Médicas, Recife, PE, Brasil. Universidade de Pernambuco Faculdade de Ciências Médicas Recife PE Brasil; 2 Universidade Federal de Pernambuco, Centro Acadêmico do Agreste, Núcleo de Ciências da Vida, Caruaru, PE, Brasil. Universidade Federal de Pernambuco Centro Acadêmico do Agreste Núcleo de Ciências da Vida Caruaru PE Brasil

**Keywords:** Malaria, Brazil, Extra-Amazon region, Epidemiological study

## Abstract

Background: Imported malaria is regularly detected in Pernambuco, a nonendemic area, highlighting the need for surveillance by healthcare professionals.

Methods: We performed a descriptive observational study of malaria cases reported in Pernambuco during 2001-2022.

Results: Most of the 350 patients were men (75.1%) and Mixed-race individuals (51.1%), with a median age of 32 years. Most patients resided in urban areas (81.1%), and 32% had primary education. Travel- and construction-related activities were the primary sources of exposure, with most infections originating in Brazil (52%) or Angola (33%). The median interval from symptom onset to testing was 10 days for Indigenous individuals, 5 days for Black individuals, and 4 days for Mixed race and White individuals. The delay was also longer for those with lower educational levels (6.5 days for primary education vs. 3 days for higher education). However, multivariate analysis showed that ethnicity, educational level, and geographic displacement were not statistically significant predictors of diagnostic delay. The cases showed a significant seasonal pattern, with a higher incidence during the first half of the year (p=0.0382). Although the annual incidence showed a slightly declining trend, this was not statistically significant (p=0.138). *Plasmodium vivax* and *P. falciparum* were the predominant species, accounting for 49.7% and 47.5% of cases, respectively.

Conclusions: Although disparities in median diagnostic times exist, multivariate analysis indicated that other complex factors are responsible for delayed diagnosis. Strengthening awareness among clinicians in nonendemic settings and ensuring timely testing are crucial for preventing severe outcomes and reducing malaria-related morbidity and mortality.

## INTRODUCTION

Malaria mainly affects tropical and subtropical regions and is considered one of the most prevalent and deadly infectious diseases in the world. The World Health Organization estimates that malaria affected approximately 263 million people and caused approximately 597,000 deaths worldwide in 2023^1^.

Malaria is endemic to some regions of Brazil, particularly the Amazon. According to the Brazilian Ministry of Health, 131,224 and 139,884 malaria cases were reported in Brazil in 2022 and 2023, respectively^2^^,^^3^, representing an increase of 6.6%. Therefore, malaria remains a public health challenge in Brazil that requires continuous efforts for its prevention, diagnosis, and treatment.

In 2019, an epidemiological alert was issued following the detection of two autochthonous cases in the municipality of Conde, Paraíba, a state neighboring Pernambuco^4^^,^^5^. Although Pernambuco is not considered an endemic area of malaria, it is naturally inhabited by *Anopheles* spp.^6^^,^^7^, including *An. aquasalis* and *An. darlingi*^5^^,^^8^. Cases of malaria were also reported in the border states of Pernambuco, Bahia, and Piauí^9^.

In Pernambuco, malaria cases were exclusively imported. As it is not an endemic area, the diagnosis may be delayed, thereby increasing the risk of a worse prognosis. We aimed to evaluate and analyze the clinical and epidemiological profiles of individuals diagnosed with malaria in Pernambuco between 2001 and 2022. Furthermore, we sought to evaluate the response time between symptoms and treatment, in addition to the parasitological profiles of the cases.

## METHODS

This observational descriptive study was performed using data from malaria cases reported in the State of Pernambuco from 2001 to 2022. Epidemiological and clinical data were obtained from the Notifiable Diseases Information System (SINAN), as provided by the Pernambuco State Health Department. In Brazil, all malaria cases must be reported. 

The following data were analyzed: sex, age, ethnicity, educational level, main activity in the last 15 days, municipality and area of residence, symptoms and date of symptom onset, place of probable infection, date of malaria diagnosis, *Plasmodium* spp. identified, parasitic load, and therapeutic regimen for malaria. Ethnicity was categorized into five groups based on the official Brazilian Institute of Geography and Statistics classification system as follows: White, Black, Pardo (Mixed race), Asian, and Indigenous.

Categorical variables were expressed as n (%) with 95% confidence intervals (CIs). Categorical variables were compared using the chi-square test (p<0.05). Continuous variables were tested for normality using the Shapiro-Wilk test and compared using the Mann-Whitney U test when the distribution was non-normal. To evaluate the association among delayed diagnosis, patient characteristics, and potential confounding factors, a multivariate linear regression model was used, which included ethnicity, educational level, and geographical displacement. The temporal and spatial distributions of malaria cases were illustrated using R software (version 4.5.1; R Core Team, 2025). A linear regression model was applied to assess the long-term trend in annual case counts, and the Mann-Whitney U test was used to evaluate seasonal patterns. All the statistical analyses were performed using Stata version 14 (StataCorp, College Station, TX, USA). This study was approved by the Research Ethics Committee of the Oswaldo Cruz University Hospital of the University of Pernambuco (CAAE 72890823.2.0000.5192).

## RESULTS

**FIGURE 1:**
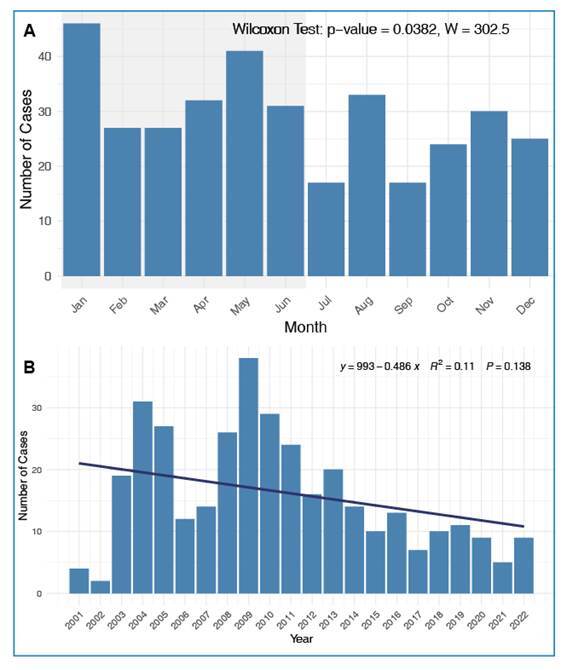
Temporal analysis of confirmed malaria cases reported in Pernambuco from 2001 to 2022. **A:** Distribution of malaria cases by month of notification. **B:** Temporal trend of malaria cases per year.

Between 2001 and 2022, 350 confirmed malaria cases were reported in Pernambuco, averaging 15.9 cases annually. The years 2009 and 2004 had the highest number of cases (38 and 31 cases, respectively). The temporal distribution demonstrated a statistically significant seasonal pattern, with a higher incidence during the first half of the year (p = 0.0382). Analysis of annual incidence showed a slight but not statistically significant declining trend (R^2^ = 0.11; p = 0.138) (Figures 1A and 1B).

Most cases occurred in men (n = 263, 75.1%) and Mixed-race individuals (n = 179, 51.1%). The median patient age was 32 years (interquartile range [IQR], 26-40 years), ranging 0-80 years. A notable proportion had 1-8 years of schooling (112, 32%), followed by those with ≥12 years (65, 18.6%) (Table 1). Most patients resided in Brazil (345, 98.6%), with 92.2% living in Pernambuco and 85.8% living in urban areas.

Three cases occurred in children aged <1 year of age in 2004, 2007, and 2009. All patients were symptomatic with low parasitemia from *P. falciparum*. The patients included two females and one male, representing Indigenous, White, and unknown ethnicities. The time from symptom onset to testing ranged 7-9 days. In one case, the probable location of the infection was documented as Amapá, Brazil.

**TABLE 1:** Epidemiological characteristics of confirmed malaria cases reported in Pernambuco from 2001 to 2022.

Variable (n=350)	n	%	95%CI
**Sex**			
Female	87	24.9	20.6 - 29.7
Male	263	75.1	70.3 - 79.4
**Age in years**			
0 - 17	24	6.9	4.6 - 10.0
18 - 30	123	35.1	30.3 - 40.3
31 - 50	168	48	42.8 - 53.3
50 - 70	31	8.9	6.3 - 12.3
71 or more	4	1.1	0.4 - 3.0
**Ethnicity**			
White	69	19.7	15.9 - 24.2
Black	27	7.7	5.3 - 11.0
Mixed-race individuals	179	51.1	45.9 - 56.4
Asian	10	2.9	1.5 - 5.2
Brazilian indigenous	17	4.9	3.0 - 7.7
No information	48	13.7	10.5 - 17.8
**Years of schooling**			
No schooling	2	0.6	0.1 - 2.3
1 - 8	112	32.0	27.3 - 37.1
9 - 11	62	17.7	14.0 - 22.1
12 or more	65	18.6	14.8 - 23.0
No information	104	29.7	25.1 - 34.7
Not applicable	5	1.4	0.6 - 3.4
**Area of residence**			
Urban	284	81.1	76.7 - 84.9
Rural	47	13.4	10.2 - 17.4
No information	19	5.4	3.5 - 8.4
Main activity in the last 15 days* (n=254)			
Agriculture	6	2.4	1.1 - 5.2
Livestock	3	1.2	0.4 - 3.6
Domestic	1	0.4	0.1 - 2.8
Tourism	13	5.1	3.0 - 8.6
Artisanal mining *(garimpo)*	3	1.2	0.4 - 3.6
Plant exploration	2	0.8	0.2 - 3.1
Construction of roads or dams	19	7.5	4.8 - 11.5
Mining	5	2.0	0.8 - 4.7
Traveler	46	18.1	13.8 - 23.4
Others	113	44.5	38.4 - 50.7
Driver	4	1.6	0.6 - 4.1
No information	39	15.4	11.4 - 20.4

* Information on recent activities was only incorporated into notification forms from 2007 onwards. **CI:** confidence interval.

In 2004, Pernambuco reported 31 malaria cases (18 male and 13 female) with a median age of 30 years (IQR 22-44). Of these, 14 (48.3%) were Indigenous and 6 (20.7%) were Mixed-race. The three cases in individuals aged <18 years were exclusively among Indigenous people (aged 1, 3, and 15 years). The species was confirmed in 24 cases; *P. falciparum* was the most common (17, 71%), followed by *P. vivax* (6, 25%), and one mixed infection (*P. falciparum* and *P. vivax*). Of the 14 Indigenous people, 11 had a confirmed cause (all caused by *P. falciparum*). Six *P. vivax* cases were treated with chloroquine and primaquine, whereas *P. falciparum* and mixed infections were treated with mefloquine and primaquine. The treatment regimen was not specified in either case.

Thirty-eight cases were reported in 2009. Most patients were male (84%) and of Mixed race (60.5%), with a median age of 31 years (IQR 26-36). The probable location of infection was specified in 11 cases, with 10 originating from the Amazon region. The most common cause was *P. falciparum* (26, 70.3%), followed by *P. vivax* (10, 27%), and one (2.7%) mixed infection. While most *P. vivax* cases were treated with chloroquine and primaquine, most *P. falciparum* cases had their treatment listed as “other regimen” without specific drugs.

The most common activities reported 15 days before infection were travel (46 cases, 18.1%) and road or dam construction (19 cases, 7.5%). Notably, 13 cases (5.1%) were specifically categorized as “tourism” (Table 1). This suggests that a substantial proportion of travel cases were for other purposes, such as employment or business, even if they were not explicitly stated in the notification form.

**FIGURE 2:**
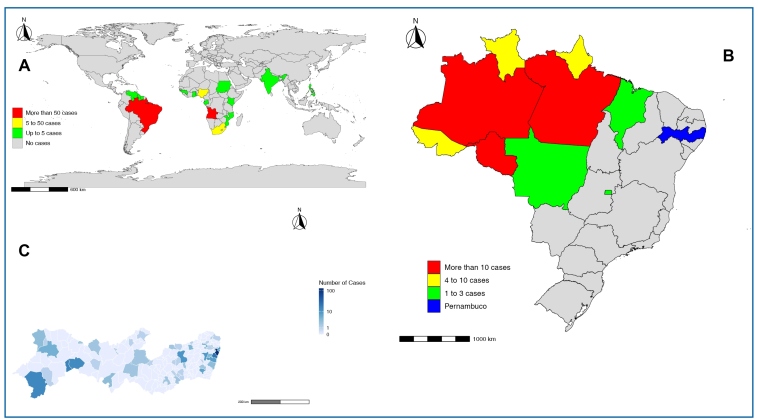
Geographic distribution of confirmed malaria cases reported in Pernambuco from 2001 to 2022. **2A:** Probable country of infection. **2B:** Probable Brazilian state of infection. **2C:** Distribution of confirmed cases by municipality of residence in the state of Pernambuco.

The majority of cases originated in Brazil (152, 51.7%), followed by Angola (98, 33.3%) and South Africa (10, 3.4%) (Figure 2A). In Brazil, most infections occurred in Amazonas (39, 25.1%), Rondônia (37, 23.9%), and Pará (34, 21.9%) (Figure 2B). Notably, 17 (11%) cases were reported to originate in Pernambuco, which likely represents a data entry error, given the state’s nonendemic status and the lack of autochthonous cases. The distribution of malaria cases by the municipality of residence in Pernambuco, Brazil, is shown in Figure 2C.

Approximately 94% of the patients presented with symptoms at the time of notification. However, the notification form included only descriptive symptom information until 2006. In the subset of cases with this information, most patients presented with fever (96.7%), chills (86.8%), and sweats (65.9%) (Table 2). After 2006, the forms only recorded the presence or absence of symptoms, with symptomatic individuals accounting for 239 (94.1%) cases from 2007 to 2022. 

**TABLE 2:** Clinical findings, *Plasmodium* species identified, and parasitic load of confirmed malaria cases reported in Pernambuco from 2001 to 2022.

Variable	n	%
**Presence of symptoms (n=349)**		
Yes	330	94.6
No	19	5.4
Symptoms * (n=95)		
Fever	88	96.7
Chills	79	86.8
Sweating	60	65.9
Headache	22	24.2
Myalgia	17	18.7
Nausea/vomiting	9	9.9
Epistaxis	1	1.1
Jaundice	1	1.1
***Plasmodium* species (n=326)**		
P. vivax	162	49.7
P. falciparum	155	47.5
*P. vivax* and *P. falciparum*	8	2.4
P. ovale	1	0.3
**Parasitic load (n=315)**		
Less than ½ + (less than 200/mm^3^)	46	14.6
½ + (200-300/mm^3^)	55	17.5
1 + (301-500/mm^3^)	32	10.2
2 + (501-10,000/mm^3^)	103	32.7
3 + (10,001-100,000/mm^3^)	67	21.3
4 + (100,000 or more/mm^3^)	12	3.8

* Information describing symptoms was included only until 2006. After this year, the notification form recorded only the presence or absence of symptoms, without describing them.

The median time from symptom onset to malaria testing was 4 days (IQR 2-9 days), with case notifications occurring a median of 5 days (IQR 2-10 days) after symptom onset. However, no delay was noted between the test and treatment, as the therapy was initiated on the same day the diagnosis was confirmed.

Stratification of the median time from symptom onset to malaria testing by ethnicity revealed a significant difference (p = 0.0035). A *post-hoc* analysis showed that the diagnostic time for Indigenous individuals (median of 10 days, IQR 8-12) was significantly longer than that for White (p_adj_ = 0.0047), Mixed-race (p_adj_ = 0.0178), and Asian (p_adj_ = 0.0053) individuals. No statistically significant differences were observed between the other groups. For Black individuals, the median was 5 days (IQR 3-10 days), Mixed-race individuals had a median of 4 days (IQR 2-9 days), White individuals had a median of 4 days (IQR 2-7 days), and Asian individuals had a median of 1 day (IQR 1-4 days).

The median time from symptom onset to testing differed significantly across educational levels (p < 0.001). A Dunn's test revealed that individuals with ≥12 years of study had a significantly shorter diagnostic time (3 days, IQR 1-5) compared to those with 1-8 years (6.5 days, IQR 3-12.5; p_adj_ < 0.001) and 9-11 years (5 days, IQR 3-10; p_adj_ = 0.0003). In contrast, no significant differences in diagnostic time were observed according to sex (p = 0.293) or age (p = 0.202).

We conducted a linear regression model that included ethnicity, educational level, and displacement (discrepancy between the municipality of notification and residence). After adjusting for displacement, ethnicity and educational level did not maintain a statistically significant association with delayed diagnosis. Additionally, geographic displacement was not a significant predictor of delayed diagnosis (p = 0.479).

Most infections were caused by *P. vivax* (n = 162, 49.7%) or *P. falciparum* (n = 155, 47.5%). Mixed infections of both species accounted for eight cases (2.4%), whereas a single case (0.3%) was attributed to *P. ovale* (Table 2). This *P. ovale* case, identified in 2022, was of a 21-year-old Black man who had recently arrived from Gabon. The diagnosis was made 4 days after symptom onset, and the patient, with a parasite load of two crosses (2-20 parasites per field or 501-10,000 parasites per cubic millimeter), was treated with chloroquine and primaquine.

Regarding the parasite load, most cases were classified as having two crosses (Table 2). Most *P. vivax* cases had two crosses, whereas *P. falciparum* cases were more frequently classified as having three crosses (10,001-100,000 parasites per cubic millimeter). This observation was confirmed statistically, with the highest parasite loads (three or four crosses) being significantly more frequent in *P. falciparum* infections (p = 0.001) (Table 3).

**TABLE 3:** Relationship between parasitemia and the parasite species in confirmed malaria cases in Pernambuco from 2001 to 2022.

***Plasmodium* species**	***P. vivax* (n=154)**		***P. falciparum* (n=151)**		***P. vivax* + *P. falciparum* (n=7)**		***P. ovale* (n=1)**	
Parasitic load	n	%	n	%	n	%	n	%
Less than ½+ (less than 200/mm^3^)	22	14.3	24	15.9	0	0	0	0
½+ (200 - 300/mm^3^)	18	11.7	35	23.2	2	28.6	0	0
1+ (301 - 500/mm^3^)	15	9.7	15	9.9	2	28.6	0	0
2+ (501 - 10,000/mm^3^)	69	44.8	29	19.2	3	42.9	1	100
3+ (10,001 - 100,000/mm^3^)	29	18.8	37	24.5	0	0	0	0
4+ (100,000 or more/mm^3^)	1	0.7	11	7.3	0	0	0	0

We analyzed 293 notifications of the prescribed therapeutic regimen. A significant proportion of cases (112, 38.2%) were categorized under “other schemes” without further specification. For *P. vivax* cases (n = 146), chloroquine plus primaquine was the predominant treatment (104, 71.2%), with 30 cases (20.5%) falling under “other schemes.” In contrast, a high percentage of *P. falciparum* cases (n = 138) were reported as “other schemes” (79, 57.2%), while 31 cases (22.4%) were treated with mefloquine plus primaquine. In eight mixed infection (*P. vivax* and *P. falciparum*), “other schemes” were noted in three cases (37.5%), while chloroquine plus primaquine and mefloquine plus primaquine were each used for two cases (25%). One patient with *P. ovale* infection was treated with chloroquine and primaquine.

## DISCUSSION

Between 2001 and 2022, 350 malaria cases were confirmed in Pernambuco, averaging 15.9 cases per year, with case spikes in 2004 and 2009. The significant seasonal pattern with cases concentrated in the first half of the year may be associated with travel patterns. We hypothesized that travelers visiting endemic areas during the end-of-year holidays (December and January) return to Pernambuco and develop symptoms shortly thereafter. This finding underscores the importance of maintaining a high index of clinical suspicion among healthcare professionals in nonendemic regions, especially during holidays.

Temporal analysis of annual malaria incidence showed no statistically significant trends. This finding suggests that annual fluctuations in case numbers are influenced more by factors other than the simple passage of time. Although a slight decrease was visually observable, the lack of statistical significance indicates that the variations are more likely to be driven by episodic events or by specific environmental, social, and public health determinants that warrant further investigation.

Our observation of a case spike in Pernambuco in 2004 aligned with a national increase in malaria cases across 16 extra-Amazonian states during the same period. This increase was also seen in Piauí (from 26 to 144 cases) and Espírito Santo (from 87 to 173 cases)^10^. This surge in the extra-Amazonian regions, along with an increasing trend in the Amazon^11^, suggests a plausible link with the movement of workers. Residents from nonendemic areas such as Pernambuco frequently travel for temporary work in malaria-endemic areas, which could be a key factor in our findings.

Although the overall number of cases in the extra-Amazonian region decreased in 2009, case numbers rose in São Paulo and Paraná^12^. The simultaneous increase observed in Pernambuco in the present study cannot be directly explained by a single factor. However, the introduction of the Laboratory Environment Manager system in 2009, a computerized system that optimized and improved the diagnostic quality of the public health system, may have led to an improvement in case notifications^13^. This suggests that our observed increase may reflect improved data capture rather than a pure increase in the number of cases.

In our study, all cases were classified as imported, that is, an infection acquired outside the area where it was diagnosed^14^, a finding consistent with Pernambuco's nonendemic status. Consistent with other Brazilian studies, our results underscore travel as the main activity associated with infection^15^^,^^16^, highlighting the critical need for clinicians to consider malaria in patients with relevant travel histories, to ensure prompt diagnosis and management^17^^-^^20^.

The epidemiological profile of cases in Pernambuco aligns with a recent report by the Brazilian Ministry of Health on malaria cases in extra-Amazonian areas^15^, with most cases occurring in adults, males, and individuals of Black or Mixed-race ethnicity. Consistent with the same report, our findings confirmed Angola as the primary country of origin for imported malaria cases in Brazil. This epidemiological link is strongly supported by the historical relationship and the significant migratory flow between the two countries^21^^,^^22^.

In nonendemic areas, rapid diagnostic tests (RDTs) are recommended because they are easy to use and do not require specialized personnel to interpret slides. However, clinicians must be aware of their limitations such as the potential for false-negative results. This is particularly relevant for *P. falciparum* infections, which can lead to severe outcomes^23^^-^^25^. Consequently, in the presence of a strong epidemiological link, such as recent travel to an endemic area, malaria should be suspected even after a negative RDT result. In these cases, a confirmatory parasitological examination using a thick smear is crucial. Moreover, as patients may not always realize the importance of this information, actively inquiring about their recent travels is crucial. This aspect of anamnesis is often overlooked during training of health professionals and students in areas where malaria is not endemic.

The high proportion of parasitemia observed was associated with *P. falciparum* infection, which is consistent with the established findings^26^. This is especially important in nonendemic settings, such as Pernambuco, where the population lacks acquired immunity and is vulnerable to severe outcomes, for which high parasitemia is a strong predictor^16^^,^^26^^,^^27^. However, it is crucial to contextualize the parasite load within the biology of each species. *P. falciparum* has the unique ability to infect blood cells of all ages, resulting in exponential replication and a high parasitic load. This capability is in contrast with the biology of *P. vivax*, which has a preferential tropism for reticulocytes^28^. This highlights the critical need for a timely and correct diagnosis, followed by fast and suitable therapy.

Malaria and dengue are the main vector-borne diseases, and both are endemic to tropical areas such as Africa^29^ and Brazil^30^. Similar initial symptoms can delay diagnosis^20^^,^^29^^,^^31^. A study in the Amazon found that among 1,578 patients with febrile syndrome, 11% had malaria and 37% had dengue. In addition, 2.8% of patients had malaria-dengue coinfection^32^. Therefore, during dengue epidemics, which are frequent events in Pernambuco, considering malaria in the differential diagnosis is crucial to improve patient prognosis^33^^-^^35^.

The data from the notification forms did not include information on patient outcomes, such as hospitalization, death, or recovery. This information is particularly important considering that individuals affected by these diseases in nonendemic regions tend to have a worse prognosis. A comparison of malaria lethality in 2019 between the Amazon and extra-Amazon regions of Brazil revealed striking differences. The lethality rate in the extra-Amazon region was 123 times higher, which may be attributed to delays in diagnosis and treatment^7^. Furthermore, the population in nonendemic areas lacks the acquired clinical immunity common in endemic regions^36^^,^^37^, making them more vulnerable to severe disease and death.

We also found inconsistencies in the notification of prescribed treatments, which limited our analysis. From 2001 to 2006, forms included a field for “other schemes;” however, the specific regimens were seldom documented. A standardized, single-entry treatment field implemented from 2007 onwards facilitated the analysis. Despite this improvement, the quality of prescribed treatment notifications remained limited throughout the study period.

For cases caused by *P. vivax*, most treatments (71%) adhered to standard protocols. In contrast, for *P. falciparum* infections, only 22% of the records described a regimen that included an active drug (mefloquine), whereas 57% were categorized as an unspecified “other scheme.” Of particular concern, contrary to treatment recommendations, the chloroquine plus primaquine regimen was prescribed for seven cases of *P. falciparum* and two mixed (*P. vivax* and *P. falciparum*) infections. This noncompliance, which has also been observed in endemic regions of Brazil^7^, suggests a persistent lack of knowledge among healthcare professionals regarding malaria management.

A key finding in this nonendemic setting was the prompt initiation of treatment, which began on the same day that the diagnosis was confirmed. This is crucial to prevent disease progression and reduce the risk of further transmission. However, while our results showed a rapid diagnostic and treatment pathway, they also revealed a significant delay in access to care for Indigenous and Black patients, which is a critical limitation of the local health system.

Historically, Black, Mixed-race, and Indigenous populations in Brazil have had limited access to healthcare services. The recent coronavirus disease 2019 (COVID-19) pandemic has led to this issue^38^. While our initial analyses revealed significant associations between ethnicity, educational level, and diagnostic delay, multivariate analysis did not support this conclusion. This suggests that these associations were mediated by confounding factors, as the complexity of healthcare access was not fully captured by our variables. The fact that travel between municipalities was not a significant predictor of delay contrasts with the common belief that geographical distance is the main barrier. This may indicate that other factors such as socioeconomic and cultural barriers or local transportation logistics underlie this disparity. Therefore, the reasons for these inequalities are multifaceted and require further investigation using more detailed data.

The persistent threat of imported malaria cases in nonendemic areas such as Pernambuco underscores its susceptibility to disease reintroduction. The 2019 outbreak in the neighboring state of Paraíba^5^, in a municipality 100 km from Recife, the capital of Pernambuco illustrates this risk. The combination of imported cases, high population mobility, and the presence of competent vectors creates a scenario involving both vulnerability and receptivity. These factors highlight the urgent need for a robust surveillance vector control program to prevent the re-establishment of local transmission. Maintaining a high index of clinical suspicion and ensuring prompt diagnostic testing are crucial for patient management and broader public health goal of preventing disease re-establishment in previously eliminated areas. Framing these actions within a one-health perspective reinforces the need for integrated approaches that consider human mobility, environmental changes, and vector ecology as interconnected drivers of malaria risk^39^.

## Data Availability

Research data is only available upon request.
